# The benefit of augmenting open data with clinical data-warehouse EHR for forecasting SARS-CoV-2 hospitalizations in Bordeaux area, France

**DOI:** 10.1093/jamiaopen/ooac086

**Published:** 2022-10-20

**Authors:** Thomas Ferté, Vianney Jouhet, Romain Griffier, Boris P Hejblum, Rodolphe Thiébaut, the Bordeaux University Hospital Covid-19 crisis task force, Isabelle Faure, Philippe Revel, Eric Tentillier, Jean-Michel Dindart, Didier Gruson, Olivier Joannes-Boyau, Jean-Marie Denis Malvy, Thierry Pistone, Didier Neau, Duc Nguyen, Marie-Edith Lafon, Mathieu Molimard, Thierry Schaeverbeke, Nicolas Grenier, Nathalie Salles, Francois Rouanet

**Affiliations:** CHU Bordeaux, Service d’Information Médicale, Place Amélie Raba Léon, Bordeaux, France; INRIA Bordeaux Sud Ouest, équipe SISTM, Talence, France; Univ. Bordeaux, Centre Inserm Bordeaux Population Health, UMR 1219, 146 rue Léo Saignat, Bordeaux, France; CHU Bordeaux, Service d’Information Médicale, Place Amélie Raba Léon, Bordeaux, France; Univ. Bordeaux, Centre Inserm Bordeaux Population Health, UMR 1219, 146 rue Léo Saignat, Bordeaux, France; CHU Bordeaux, Service d’Information Médicale, Place Amélie Raba Léon, Bordeaux, France; Univ. Bordeaux, Centre Inserm Bordeaux Population Health, UMR 1219, 146 rue Léo Saignat, Bordeaux, France; INRIA Bordeaux Sud Ouest, équipe SISTM, Talence, France; Univ. Bordeaux, Centre Inserm Bordeaux Population Health, UMR 1219, 146 rue Léo Saignat, Bordeaux, France; CHU Bordeaux, Service d’Information Médicale, Place Amélie Raba Léon, Bordeaux, France; INRIA Bordeaux Sud Ouest, équipe SISTM, Talence, France; Univ. Bordeaux, Centre Inserm Bordeaux Population Health, UMR 1219, 146 rue Léo Saignat, Bordeaux, France

**Keywords:** SARS-CoV-2, forecasting, electronic health records, Data Warehouse, Machine Learning

## Abstract

**Objective:**

To develop an accurate regional forecast algorithm to predict the number of hospitalized patients and to assess the benefit of the Electronic Health Records (EHR) information to perform those predictions.

**Materials and Methods:**

Aggregated data from SARS-CoV-2 and weather public database and data-warehouse of the Bordeaux hospital were extracted from 2020-05-16 to 2022-01-17. The outcomes were the number of hospitalized patients in the Bordeaux Hospital at 7 and 14 days. We compared the performance of different data sources, feature engineering and machine learning models.

**Results:**

During the period of 88 weeks, 2561 hospitalizations due to COVID19 were recorded at the Bordeaux Hospital. The model achieving the best performance was an elastic-net penalized linear regression using all available data with a median relative error at 7 and 14 days of 0.136 [0.063; 0.223] and 0.198 [0.105; 0.302] hospitalizations, respectively. Electronic health records (EHRs) from the hospital data-warehouse improved median relative error at 7 and 14 days by 10.9 and 19.8%, respectively. Graphical evaluation showed remaining forecast error was mainly due to delay in slope shift detection.

**Discussion:**

Forecast model showed overall good performance both at 7 and 14 days which were improved by the addition of the data from Bordeaux Hospital data-warehouse.

**Conclusion:**

The development of hospital data-warehouse might help to get more specific and faster information than traditional surveillance system, which in turn will help to improve epidemic forecasting at a larger and finer scale.

**LAY SUMMARY:**

The objective of this work was to develop a forecast algorithm to predict the number of hospitalized patients at Bordeaux Hospital. In addition, we assessed the benefit of the Electronic Health Records (EHRs) information to perform those predictions.

To perform this task, we used data between 2020-05-16 and 2022-01-17 from national database on SARS-CoV-2 epidemics, public database on weather and the data-warehouse of the Bordeaux hospital. The outcomes were the number of hospitalized patients in the Bordeaux Hospital at 7 and 14 days.

During the period of 88 weeks, 2561 hospitalizations due to COVID19 were recorded at the Bordeaux Hospital. The best model had an error of 13.6% at 7 days and 19.8% at 14 days. EHRs from the hospital data-warehouse improved the performance by 10% at 7 days and 20% at 14 days. Graphical evaluation showed remaining forecast error was mainly due to delay in slope shift detection.

Forecast model showed overall good performance which were improved by the addition of EHRs data. The development of hospital data-warehouse might help to get more specific and faster information than traditional surveillance system, which in turn will help to improve epidemic forecasting at a larger and finer scale.

## BACKGROUND AND SIGNIFICANCE

Since the end of 2020, millions of SARS-CoV-2 cases have been reported worldwide.[1–3] This pandemic has had a major impact on health care with an increase of the hospitalizations leading to modifications of the organization of care and to unprecedented population lockdowns to avoid health care system saturation.[4–6] The ability to anticipate the evolution of the epidemic at a local level is critical to manage the health care system.

To achieve this goal, several forecasting algorithms have been proposed.[7,8] None was fully satisfactory. Cramer et al. [8] compared different approaches, including regression, compartmental, ensemble, deep-learning, to forecast the number of death related to covid-19 in the United States. Best models included ensemble, deep learning and several compartmental methods. They all used epidemiological data and, depending on the model, mobility and demographics data. Although the performances varied from algorithm to the other, the data used differed between best models without clear trend indicating which data would provide the best performance.

Because many factors change over time (e.g. population behaviour, government policies, vaccine coverage, virus strain), long-term forecast of COVID-19 is impossible. In France, several approaches to short-term epidemic forecast have been proposed relying on linear algebra,[9] ensemble methods[10] or neural networks.[11,12] All models aimed to forecast hospitalizations (among other things), except the one proposed by Carvalho et al., which focused on cases, ICU and deaths.[11] The data used were hospitalizations, Reverse Transcriptase Polymerase Chain Reaction (RT-PCR), Intensive Care Units (ICU), weather, mobility, vaccination, variants of concern and mask wearing policy data. The approach proposed by Mohimont et al., based on Convolutional Neural Networks seems to achieve the best results with a normalised root mean square deviation at 14 days of 3.2% compared to a mean absolute percentage error of 20% of the approach proposed by Paireau et al at the national level. However, the periods of evaluation are different (May 2021 for Mohimont et al.[12], March to July 2021 for Paireau et al.[10]) and direct comparison is difficult. In addition, graphical evaluation showed struggle to anticipate slope shift, that is a change in the dynamic of the epidemic toward an increase or a decrease of the number of cases. In addition, the performance improvement added by the different data sources was not formally evaluated. Finally, no model was satisfactory and valid enough to implement immediately.

Previous work focused mainly on national[9–12] or regional[10,12] forecast. Yet, finer granularities are needed to inform on local epidemic evolutions. Because hospitals are key actors during this pandemic and their saturation is a critical factor of sanitary policies, they are a relevant scale for local forecasting. Since November 2017, the Bordeaux University Hospital has developed a data-warehouse based on i2b2 architecture.[13] It facilitates the use of electronic health records and allows extracting detailed information of the epidemic such as the emergency units and ambulance service notes. We hypothesized that those local data should improve the forecast of the SARS-CoV-2 epidemic.

## OBJECTIVE

The objective of this work was to develop an accurate regional forecast algorithm to predict the number of hospitalized patients and to assess the benefit of the Electronic Health Records (EHR) information to perform those predictions.

## MATERIALS AND METHODS

Aggregated data from 2020-05-16 to 2022-01-17 regarding French COVID-19 epidemic were included. In order to improve forecasting, several data sources were used.

### Open data

Open data included both epidemiologic data from Santé Publique France and weather data from National Oceanic and Atmospheric Administration (NOAA) Integrated Surface Database.[14,15] Both provide department aggregated data and are daily updated.

Santé Publique France data included hospitalizations, number of RT-PCR, positive RT-PCR, proportion of positive RT-PCR, dominant variant and number of first dose vaccinated. RT-PCR data were available by age and were grouped as 0-19, 20-59 and 60 and more years old categories. Variant identification data before 2021-02-18 were not available, and majority variant before that date was assigned to wild type.

NOAA data, including temperature, wind speed, humidity and dew point, were extracted and the Predict Index for COVID-19 Climate Transmissibility – *Index PREDICT de Transmissivité Climatique de la COVID-19* – (IPTCC) was computed [15]. Missing weather data were imputed using a two steps procedure: (i) the mean value of the adjacent department was imputed; (ii) remaining missing values were imputed using last observation carried forward.

### EHR data

The Bordeaux Hospital is a large structure comprising three hospital structures taking care of nearly 250 000 hospitalized patients and 100 000 emergency consultations during 2020.[16] A data warehouse based on i2b2 structure was built in 2017.[13] This star architecture is based on a central fact table where each row represents a diagnosis, a laboratory result, a procedure, a medical observation, etc. Each fact is related to other tables with information about the patient, the visit, the provider or type of fact.[17] This structure allows for quick data queries compared to the usual siloed organizations.

To perform those queries, ontology alignment have been performed on laboratory results, and ad hoc natural language processing tools have been developed, including ROMEDI (i.e. a French drug terminology to extract drug information from text), IAMsystem (i.e. a dictionary-based approach for name entity recognition) and SmartCRF (i.e. a software to visualize and annotate EHR).[18–20] Several applied projects were performed using those tools including automatic detection of surgical site infection and transfusion associated circulatory overload.[21,22]

Thanks to those previous experiences, the Bordeaux hospital data warehouse was used, during the pandemic, to describe the current state of the epidemic at the hospital level on a daily basis. Those data were then used in the forecast model including: hospitalizations, hospital and ICU admission and discharge, ambulance service notes and emergency unit notes. Concepts related to COVID-19 were extracted from notes by dictionary-based approaches (e.g. cough, dyspnoea, covid-19). Dictionaries were manually created based on manual chart review to identify terms used by practitioners. Then, the number and proportion of ambulance service calls or hospitalization in emergency units mentioning concepts related to covid-19 were extracted. Detail of features is available in the table S1 in the supplementary material.

Due to different data acquisition mechanisms, there was a delay between the occurrence of events and the data acquisition. It was of 1 day for EHR data, 5 days for department hospitalizations and RT-PCR, 4 days for weather, 2 days for variants and 4 days for vaccination. For the training and evaluation of the model, the chosen date was the date of data availability to mimic a real-time streaming forecast.

### Statistical and machine learning models

The outcomes were the number of hospitalized patients with SARS-CoV-2 infection in the Bordeaux hospital at 7 and 14 days. Several statistical models and machine learning algorithms were compared: linear regression and Poisson regression with elastic-net penalization, random forest and Fréchet random forest (i.e. a random forest derived method able to learn directly from time series).[23] Negative predicted values were forced to 0.

### Modelling strategy

To train the model, the primary analysis used a matrix where each row corresponds to a day and each column to a feature from Gironde (i.e. the Bordeaux department) open source and Bordeaux Hospital EHR data. Other department data were not added to limit the number of features. Prediction performance were evaluated depending on the data used for the forecast, initially using only hospitalisation and RT-PCR and progressively adding SARS-CoV-2 incidence in Gironde, weather, EHRs, Vaccine and Variant data. Because of the elastic-net penalisation,[24] each day the model might select different features by shrinking beta coefficient of unimportant features to zero.

An additional analysis considered the Bordeaux Hospital as an additional unit among the other French departments. The advantage was to leverage the information from all the departments; the drawback was the impossibility to include specific information only available for the Bordeaux Hospital. Of note, the incorporation as a third level inside the Gironde department would be possible in theory but it would lead to the same restrictions and it would not be feasible in absence of other EHR data from other departments. Results from this additional analysis are available in table S2 in the supplementary material.

Several feature engineering transformations were performed. The mean, minimum and maximum value over the last 7 days were computed for each feature, as well as the first derivatives over the last 3, 7, 10 and 14 days. Features were smoothed using a local polynomial regression with a span of either 0 (i.e. no smoothing), 7, 14 or 21 days to take into account outliers and weekly variations.

### Model evaluation

The models were evaluated every day on the data available from 2020-12-01 to 2022-01-17 (i.e. data from 2020-05-16 to 2020-12-01 were used for training only). The models were trained using all prior data available at a given date *d* and the forecast of the number of hospitalizations at both 7 and 14 days after *d* was evaluated. Prediction performances were evaluated according to median absolute error (MAE) and median relative error (MRE). Median was chosen over mean because it is less sensitive to extreme values which are frequent when the observed outcome is low (i.e. a small absolute error could result in a huge relative error). Bootstrapped 95% confidence interval were provided with 500 samples (more details in the supplementary material). Graphical evaluation was also performed. Prediction intervals were estimated by bootstrap and also with an operational rule of more or less 10 and 20% of the predicted value at 7 and 14 days. For the latter, given $\widehat{hosp}$ the predicted hospitalization, the prediction interval at 7 and 14 days were $[0.9 \times \widehat{hosp}\ ; 1.1 \times \widehat{hosp}\ ]$ and $[0.8 \times \widehat{hosp}\ ; 1.2 \times \widehat{hosp}\ ]$ respectively.

## RESULTS

### Description

Relationship between predictors and hospitalizations change over time. For instance, as described in figure S1 in the supplementary material, both RT-PCR in the Bordeaux Hospital and in Gironde well anticipated hospitalizations from June 2020 to December 2020. Unfortunately, the relationship become less consistent beyond December 2020. For instance, Gironde RT-PCR are synchronous to hospitalization peak in April 2021 but do not anticipate it. In addition, the large increase of positive RT-PCR during the end of the summer 2021 is not associated with a similar increase of hospitalizations, which is probably due to vaccination. Those findings explain why the forecast of SARS-CoV-2 hospitalizations is difficult and why it is interesting to leverage different data sources to improve prediction capacity.

### Model performance

Table 1: Forecast performance by data source. In bold, the best model per forecast time frame.

| Forecast | Data [number of features] | MAE [95% CI] | MRE [95% CI] |
|---|---|---|---|
| 7 days | Hosp + RT-PCR [266] | 9·04 [6·36 ; 11·88] | 0·176 [0·116 ; 0·243] |
| 7 days | Hosp + RT-PCR + Gironde Hosp + Gironde RT-PCR [526] | 8·79 [6·41 ; 11·19] | 0·171 [0·119 ; 0·227] |
| 7 days | Hosp + RT-PCR + Gironde Hosp + Gironde RT-PCR + Weather [666] | 7·51 [4·72 ; 10·38] | 0·156 [0·097 ; 0·222] |

| | | | |
|---|---|---|---|
| 7 days | Hosp + RT-PCR + Gironde Hosp + Gironde RT-PCR + Weather + CT-scan + emergency units + ambulance service [2986] | 6·59 [3·05 ; 9·78] | 0·139 [0·065 ; 0·222] |
| **7 days** | **Hosp + RT-PCR + Gironde Hosp + Gironde RT-PCR + Weather + CT-scan + emergency units + ambulance service + Variants + Vaccine [2990]** | **6·67 [3·08 ; 9·75]** | **0·136 [0·063 ; 0·223]** |
| 7 days | Hosp + RT-PCR + Gironde Hosp + Gironde RT-PCR + Weather + CT-scan + emergency units + ambulance service + Variants + Vaccine + interaction (Vaccine x RT-PCR) [3470] | 6·12 [2·12 ; 9·89] | 0·125 [0·048 ; 0·218] |
| 14 days | Hosp + RT-PCR [266] | 15·88 [9·30 ; 21·56] | 0·334 [0·204 ; 0·435] |
| 14 days | Hosp + RT-PCR + Gironde Hosp + Gironde RT-PCR [526] | 17·21 [9·92 ; 24·99] | 0·318 [0·187 ; 0·458] |
| 14 days | Hosp + RT-PCR + Gironde Hosp + Gironde RT-PCR + Weather [666] | 11·63 [6·90 ; 17·16] | 0·242 [0·143 ; 0·339] |
| 14 days | Hosp + RT-PCR + Gironde Hosp + Gironde RT-PCR + Weather + CT-scan + emergency units + ambulance service [2986] | 9·48 [4·92 ; 15·46] | 0·194 [0·103 ; 0·304] |
| **14 days** | **Hosp + RT-PCR + Gironde Hosp + Gironde RT-PCR + Weather + CT-scan + emergency units + ambulance service + Variants + Vaccine [2990]** | **9·37 [4·85 ; 15·34]** | **0·198 [0·105 ; 0·302]** |
| 14 days | Hosp + RT-PCR + Gironde Hosp + Gironde RT-PCR + Weather + CT-scan + emergency units + ambulance service + Variants + Vaccine + interaction (Vaccine x RT-PCR) [3470] | 9·74 [4·67 ; 16·87] | 0·212 [0·097 ; 0·329] |

| | | | |
|---|---|---|---|
| 11 days | Hosp + RT-PCR + Gironde Hosp + Gironde RT-PCR + Weather + CT-scan + emergency units + ambulance service + Variants + Vaccine [2990] | 8·75 [4·13 ; 13·37] | 0·185 [0·091 ; 0·298] |
| 18 days | Hosp + RT-PCR + Gironde Hosp + Gironde RT-PCR + Weather + CT-scan + emergency units + ambulance service + Variants + Vaccine [2990] | 11·97 [5·51 ; 18·45] | 0·235 [0·115 ; 0·350] |

MAE: Median Absolute Error - MRE: Median Relative Error

Table 1 shows the forecast performance depending on the features included in the model. A simple model including the hospitalizations and the RT-PCR led to a MAE of 9·04 and MRE of 17·6%, that is a prediction up to 17·6% more or less than the observed number of hospitalization 7 days later. The addition of RT-PCR and hospitalizations from the Gironde area slightly decreased MAE at 7 days by 0·25, increased it at 14 days by 1·33 and decreased MRE by respectively 0·5% and 1·6% at 7 and 14 days. Adding weather data improved 7- and 14-days MAE by respectively 1·28 and 5·58 hospitalizations. The addition of specific EHR data from the hospital data warehouse improved the forecast at both 7 and 14 days by respectively 0.92 and 2·15 in term of MAE, and by 1·7% and 4·8% in term of MRE. As both emergency units and ambulance service data provide information about the symptomatic patients who are different from the positive patients detected by RT-PCR and the severe patients detected by hospitalization, they might help to better anticipate the evolution of the epidemic. Panel C of figure 2 supports those findings and shows that using EHR data anticipates the April peak, better forecasts the 2021-2022 winter increase and is more robust to the hospital cluster of December 2020. Last, we evaluated the information added by the number of vaccinated people and the variants distribution. Those additional data provided similar forecast performance and vaccine-RT-PCR interaction improved forecast at 7 days but decreased it at 14 days. Similar results were obtained with the additional analysis considering the Bordeaux Hospital as an additional department; results are available at table S2 in the supplementary material. Overall, we considered the model including all the data but without the vaccine – RT-PCR interaction as the best model.

Information retrieval from data-warehouse was done with a 1-day delay whereas it was a 5 days delay for hospitalizations and RT-PCR at the department level. Therefore, forecasting using data from Bordeaux Hospital data warehouse gives a 4 days advantage over usual open data. To evaluate its consequences, we compared 7 days forecast with 11 days forecast and 14 days forecast with 18 days forecast. As shown in table 1, this resulted in a MAE and MRE decrease of respectively 2·08 and 4·9% at 7 days and of respectively 2·6 and 3·7% at 14 days.

Table 2: Forecast performance depending on modelling hyper-parameters. In bold, the best model per forecast time frame.

| **Forecast** | **Feature transformation** | **Smoothing span** | **Machine-learning Model** | **MAE [95% CI]** | **MRE [95% CI]** |
|---|---|---|---|---|---|
| 7 days | Raw, Mean, Min Max, Derivative | 0 days | Linear elastic-net | 8.34 [3.13 ; 13.21] | 0.168 [0.063 ; 0.284] |
| 7 days | Raw, Mean, Min Max, Derivative | 7 days | Linear elastic-net | 8.23 [2.72 ; 13.24] | 0.169 [0.057 ; 0.284] |
| 7 days | Raw, Mean, Min Max, Derivative | 14 days | Linear elastic-net | 7.00 [2.42 ; 11.34] | 0.162 [0.057 ; 0.263] |
| **7 days** | **Raw, Mean, Min Max, Derivative** | **21 days** | **Linear elastic-net** | **6.67 [3.08 ; 9.75]** | **0.136 [0.063 ; 0.223]** |
| 7 days | Raw | 21 days | Linear elastic-net | 7.50 [0.90 ; 17.02] | 0.151 [0.021 ; 0.331] |
| 7 days | Raw, Mean, Min Max, Derivative | 21 days | Poisson elastic-net | 11.50 [5.75 ; 19.37] | 0.262 [0.144 ; 0.419] |
| 7 days | Raw, Mean, Min Max, Derivative | 21 days | Random Forest | 8.00 [4.00 ; 13.00] | 0.173 [0.088 ; 0.265] |

| | | | | | |
|---|---|---|---|---|---|
| 7 days | 14 days raw curves | 21 days | Fréchet Forest | 7.62 [3.44 ; 12.11] | 0.169 [0.084 ; 0.274] |
| 14 days | Raw, Mean, Min Max, Derivative | 0 days | Linear elastic-net | 11.84 [5.62 ; 18.43] | 0.249 [0.107 ; 0.363] |
| 14 days | Raw, Mean, Min Max, Derivative | 7 days | Linear elastic-net | 12.79 [6.78 ; 19.25] | 0.267 [0.123 ; 0.387] |
| 14 days | Raw, Mean, Min Max, Derivative | 14 days | Linear elastic-net | 10.75 [5.29 ; 16.78] | 0.217 [0.097 ; 0.344] |
| **14 days** | **Raw, Mean, Min Max, Derivative** | **21 days** | **Linear elastic-net** | **9.37 [4.85 ; 15.34]** | **0.198 [0.105 ; 0.302]** |
| 14 days | Raw | 21 days | Linear elastic-net | 12.61 [3.01 ; 25.47] | 0.306 [0.056 ; 0.580] |
| 14 days | Raw, Mean, Min Max, Derivative | 21 days | Poisson elastic-net | 15.31 [7.17 ; 25.73] | 0.370 [0.205 ; 0.552] |
| 14 days | Raw, Mean, Min Max, Derivative | 21 days | Random Forest | 13.00 [10.00 ; 18.00] | 0.269 [0.190 ; 0.357] |
| 14 days | 14 days raw curves | 21 days | Fréchet Forest | 13.52 [7.77 ; 18.49] | 0.232 [0.145 ; 0.339] |

MAE: Median Absolute Error - MRE: Median Relative Error

14 days raw curves: as Fréchet model are able to learn trends directly from curves, they are not provided with transformed features.

Models with “Raw” data (i.e no transformation) are trained on 159 features. Models trained on “Raw, Mean, Min Max, Derivative” are trained on 2990 features.

Table 2 shows the performance according to feature engineering and statistical model. Model performance were improved by smoothing and by feature transformation (i.e. mean, minimum, maximum and first derivative) of respectively 1·67 and 0·83 absolute error at 7 days and 2·47 and 3·24 absolute error at 14 days. Linear regression with elastic-net penalization outperformed random forest, Fréchet random forest and Poisson regression with elastic-net penalization. Finally, the best model was the elastic-net model using hospitalizations, RT-PCR, weather, vaccine, variant, emergency units, ambulance service data, vaccine and majority variant with a smoothing span of 21 days and the mean, min, max and first derivative feature transformation.

Figure 1 represents the feature importance of the model in term of percentage of selection over the days with asymptotic confidence intervals. At 7 days, the most important features were the ones related to hospitalization, as there is an intrinsic dynamic of hospitalizations, which is less susceptible to be influenced by external factors on such a short period. At 14 days, the most important features were related to RT-PCR in the 60+ years old group, weather data, emergency units and hospitalizations.

*Insert Figure 1 here*

*Insert figure 2 here*

Figure 2.A shows the best model predictions from 1 to 14 days. Predictions were mostly accurate except (i) in December 2020 during the hospital nosocomial cluster at Bordeaux Hospital which had a specific dynamic, (ii) in the end of March 2021 where the April decrease is anticipated 2 weeks earlier, and (iii) during the summer 2021 and the winter 2021-2022 where the forecast is overestimating hospitalizations partly because the RT-PCR increased massively whereas the hospitalizations increased moderately as depicted in figure S1. The latter might be a consequence of the vaccination campaign and the omicron spread. Figure 2.B shows the prediction intervals of the forecast using an ad hoc rule of respectively 20% and 40% prediction interval at 7 and 14 days, which has better coverage percentage than bootstrapped prediction intervals available in the supplementary material. This Figure shows that prediction intervals are mostly correct except during summer 2021 and winter 2021-2022. The former is explained by the low number of infected— which generates narrow prediction intervals — and the introduction of vaccination which biases the prediction forecast. The latter is explained by the overestimation of hospitalization rise.

### Model evolution over time

*Insert figure 3 here*

Figure 3 shows the evolution of MAE and MRE at 7 and 14 days over time. Globally, both MAE and MRE tend to be higher in December 2020, which corresponds to an intra-hospital cluster. They also increase after June 2021, which is probably related to the impact of the vaccination campaign followed by the rise of omicron variant.

Selected features by elastic-net penalization change over time. At 14 days forecast, we observe that features related to UHB positive PCR (especially in the elderly), hospitalizations, and IPTCC are frequently selected during all the period. Of note, features related to paediatric emergency are more often selected after August 2021. At 7 days forecast, the most frequently selected features are less variable over time, with the RT-PCR in the elderly, the hospitalizations and the IPTCC frequently selected. More details are available in supplementary material at figure S5 and S6.

## DISCUSSION

This work demonstrates good overall forecast ability, both in term of relative and absolute error of predictions which where both improved by the addition of weather and data-warehouse information. In addition, the intrinsic “rules” governing the epidemic evolve over time, and external interventions such as vaccination make the hospitalization time series non-stationary, which is an important challenge for a data driven approach. To mitigate this, we used an adaptive approach where the model is trained every day on historical data, which is realistic given the swiftness of penalized linear regression training. In addition, elastic-net penalization allows the model to select different features each day enhancing the adaptiveness of the model.

In their review, Rahim et al. [7] identify compartmental models and deep learning as the most common approaches to perform short term forecast, followed by machine learning and statistical learning. Formal comparisons of their performance is difficult because dates and locations vary across the reviewed articles. Table 3 summarise findings of selected related work. Cramer et al. [8] describe the COVID-19 Forecast Hub, an open platform where several academic, industry and independent groups proposed forecasting model for US SARS-

CoV-2 cases, hospitalizations and deaths, which permits head-to-head comparison. In their paper, they evaluated death forecast performance. They show that performances varied from one algorithm to another, showing no trend as to which data would consistently provide the best performance. Interestingly, the best algorithm used ensemble method, second and third best algorithms used compartmental method.

In France, forecast performance of previous studies are generally claimed to be good as depicted at table 3.[9,10,12]. However, benchmarking and comparisons is difficult due to different time period and geographical scales. Graphical evaluations help to qualitatively compare model behaviour, especially considering slope shifts, but it is not always provided [9,12] and can show difficulties to anticipate those[10]. Off note, the work proposed by Mohimont et al. [12] was able to anticipate November hospitalization slope shift at the national level.

The main strength of our study was to leverage information from different sources. The addition of weather did improve forecasting which is consistent with previous work.[25] The use of specific information from a hospital data-warehouse improved the performance both by increasing the amount of information and by updating the information faster than public data sources.

In our work, the vaccine and variants data had little effect on the performance of the model. This was partly expected because vaccine is a monotonous increasing curve and majority variant is relatively stable over time as shown in the Figure S2 in the supplementary material. In addition, there might be mismeasurement bias of variant as not every Sars-CoV-2 positive swab is tested for variant identification. Furthermore, the information added by variant and vaccine might already be captured by both the RT-PCR and hospitalization features. We also observed that RT-PCR-variant interaction deteriorated the model performance at 14 days. This was explained by a spurious positive correlation between the interaction term and the hospitalizations. Nevertheless, both vaccine and variant are linked to the SARS-CoV-2 epidemic and the inability to leverage that information in machine learning approach might call for a more mechanistic one.[25–28]

The moderate impact of the addition of Gironde data to the model might be explained by: (i) the information already being captured by the local data from the Bordeaux Hospital and (ii) the data collection being slower at the department level than in the hospital. Data consolidation (i.e. the update of prior data thanks to new information) was not taken into account because of lack of availability of data versioning. For instance, hospitalization related to Sars-Cov-2 is sometimes identified in the EHR through billing codes in International Classification of Disease, 10th revision, which are only available at the end of the sojourn. This also might have biased the performance evaluation.

**Table 3: Summary of discussed forecasting methods.**

| Study | Location | Data* | Methods | Outcome** | Performance |
|---|---|---|---|---|---|
| Paireau et al. [10] | France | EDMW | Ensemble | H at 14 days | MAPE at 14 days : 20% |
| Mohimont et al. [12] | France | EDM | Convolutional Neural Networks | H at 14 days | Normalised root mean square deviation at 14 days : 3.2% |
| Pottier et al. [9] | France | E | Linear algebra | H at 14 days | Average relative error : 12% |
| Carvalho et al. [11] | France | E | Neural Networks | C, D and ICU at 14 days | MAPE : C 4.13%, D 10.26%, ICU 0.92% |
| Cramer et al. [8] | US | EDM | Ensemble (best) | D at 4 weeks | RMAEvB : 0.66 |
| | | E | UMass-MechBayes, Bayesian compartmental model (second best) | D at 4 weeks | RMAEvB : 0.67 |

| | | | |
|---|---|---|---|
| E | Karlen-pypm, discrete time compartmental model (third best) | D at 4 weeks | RMAEvB : 0.70 |

* : E (Epidemiological), D (Demographic), M (Mobility), W (Weather)

** : H (Hospitalisations), D (Death due to COVID-19), ICU (Intensive Care Units), C (Cases)

Mean absolute percentage error (MAPE)

Relative Mean Absolute Error compared to a baseline model of constant prediction (RMAEvB)

Linear regression with elastic-net penalization outperformed other more complex models such as Fréchet or classical random forest. This might be explained by (i) a linear relationship between the features and (ii) the difficulty for random forest to extrapolate features relationship outside of the training set space that a linear regression can handle more naturally.

This work outlined the advantage of adding information from the EHRs to improve the forecast, especially at 14 days and during November and December 2021 as depicted at figure 2, panel C. This was expected because the EHRs provide additional information about the local evolution of the pandemic through the emergency units and the ambulance service data. Those data inform on the more specific population of covid-19 symptomatic patients who are different from the overall covid-19 patients detected by RT-PCR and the severe covid-19 patients who are hospitalised.

Figure 3 outlined that model performance is not homogeneous. Relative error was generally higher when the number of hospitalisations was low (summer 2021, November 2021) which is expected because the denominator is lower during those periods. Absolute error was higher during December 2020, which is expected because the model did not learn on many observations yet, and there was a hospital cluster at this period. It was also higher during the summer 2021 that might be explained by the beginning of vaccine campaign effect.

Although the model performance can be considered as good, there are some limitations. First, performance tends to deteriorate when there is a sudden change of the hospitalisations dynamic. For instance, in February and March 2021, the decrease and the increase of the hospitalisations were not well anticipated by the model. Second, change of the infectiousness of the virus either due to a mutation or vaccine were not immediately learned by the model. This might explain the overestimate during summer 2021 and it might occur again in the future. Third, the model used for this task is a linear regression and it might not capture complex relations. As discussed before, random forest did not improve forecast but other machine learning methods such as reservoir computing may.[29]

The model is currently used in the Bordeaux University Hospital on a daily basis to anticipate the evolution of the number of covid-19 hospitalisations. The forecast is used in conjunction with other indicators (number of hospitalisations, RT-PCR, emergency unit's workload, etc.) and is discussed with both clinicians and public health experts. In our experience, the forecast is particularly informative to anticipate when a local peak is reached and the hospitalisations will decrease or the arrival of a new wave.

## CONCLUSION

This work highlights the advantage of leveraging several different data sources to improve forecast accuracy. The development of hospital data-warehouse might help to get more specific and faster information than a traditional surveillance system, which in turn will help to improve epidemic forecasting at a larger and finer scale.

## ACKNOWLEDGMENTS

Preprint of this paper is available at SSRN: https://ssrn.com/abstract=4071506 or http://dx.doi.org/10.2139/ssrn.4071506. Code of this paper is available on https://github.com/thomasferte/PredictCovidOpen.git. Computer time for this study was provided by the computing facilities of the MCIA (Mésocentre de Calcul Intensif Aquitain). This work has been partly supported by Inria, Mission COVID19, GESTEPID project and Nouvelle Aquitaine regional funding (Prediction territorial COVID N°1333140). We thank Louis CAPITAINE and Robin GENUER for their help with Fréchet random forest. We thank the Bordeaux University Hospital Covid-19 crisis task force for their help with the data curation and the interpretation: Isabelle FAURE, Philippe REVEL, Eric TENTILLIER, Jean-Michel DINDART, Didier GRUSON, Olivier JOANNES-BOYAU, Jean-Marie Denis MALVY, Thierry PISTONE, Didier NEAU, Duc NGUYEN, Marie-Edith LAFON, Mathieu MOLIMARD, Thierry SCHAEVERBEKE, Nicolas GRENIER, Nathalie SALLES, Francois ROUANET.

## COMPETING INTERESTS

We declare no competing interests.

## FUNDING

This work has been partly supported by Inria, Mission COVID19, GESTEPID project and Nouvelle Aquitaine regional funding (Prediction territorial COVID N°1333140).

## ETHICAL STATEMENT

No ethics committee approval was needed for this work as data used for modelling were aggregated.

## AUTHORSHIP AND CONTRIBUTIONS

TF contributed to the data curation, formal analysis, software and writing - original draft. VJ contributed to conceptualization, data curation, supervision, funding acquisition, writing - original draft. RG contributed to data curation, software, writing - original draft. BH contributed to conceptualization, methodology, supervision, writing - original draft. RT contributed to conceptualization, methodology, supervision, funding acquisition, writing - original draft.

## DATA SHARING

Open data used for this study are available on data.gouv.fr[14] and ncei.noaa.gov.[15] EHR data used for this study are available upon request. Data available from the Dryad Digital Repository: https://doi.org/10.5061/dryad.hhmgqnkkx. Code available from github: https://doi.org/10.5281/zenodo.6595011.

## FIGURES

**Figure 1: Top 20 features importance with 95% confidence interval of the best model. As model is re-trained every day and is elastic-net penalized, each dot represents the proportion of days for which the feature is selected. SAMU = French ambulance service**

**Figure 2: Forecast of best model at 7 and 14 days. Panel A shows the best model forecast from 1 to 14 days. Each string corresponds to the forecast from one (yellow) to 14 days (purple) at each day. Panel B shows the 20% prediction intervals at 7 days and 40% at 14 days. Panel C compares the forecast without EHR data (i.e. Hospitalizations + RT-PCR + Gironde Hospitalizations + Gironde RT-PCR + Weather + Variant + Vaccine).**

**Figure 3: Evolution of MAE and MRE over time at 7 and 14 days. Each dot corresponds to the median absolute error of the corresponding month with the 95% bootstrapped confidence interval.**

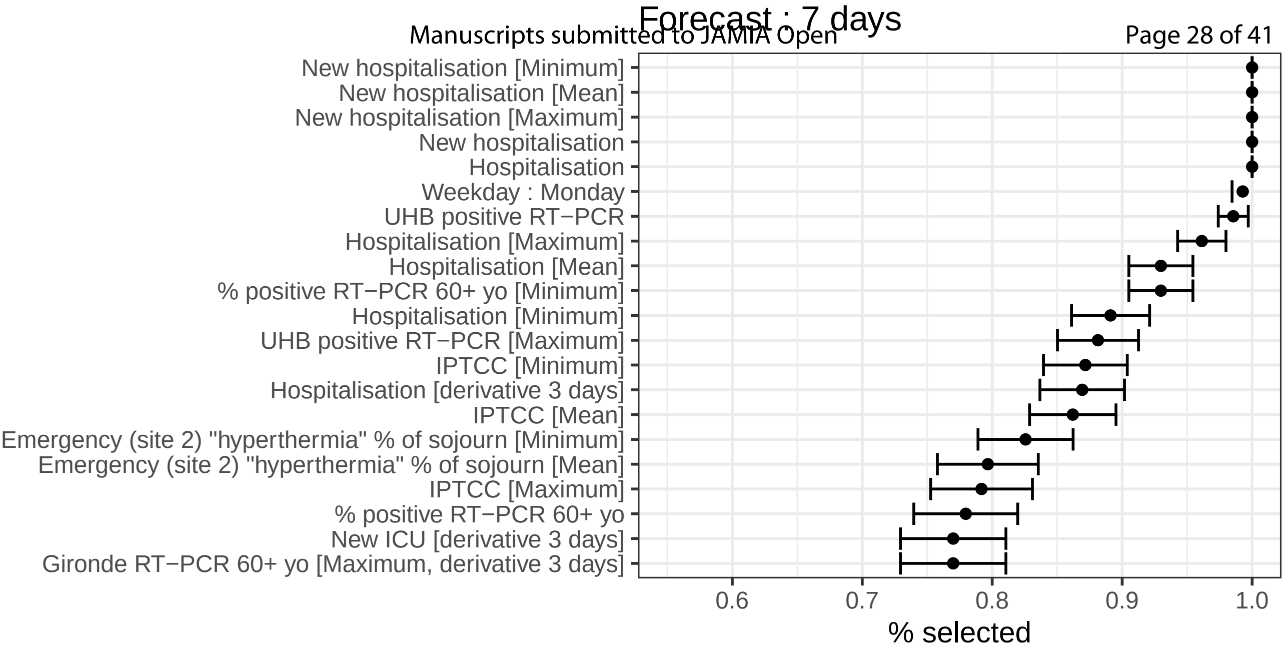

Forecast : 7 days
New hospitalisation [Minimum]
New hospitalisation [Mean]
New hospitalisation [Maximum]
New hospitalisation
Hospitalisation
Weekday : Monday
UHB positive RT−PCR
Hospitalisation [Maximum]
Hospitalisation [Mean]
% positive RT−PCR 60+ yo [Minimum]
Hospitalisation [Minimum]
UHB positive RT−PCR [Maximum]
IPTCC [Minimum]
Hospitalisation [derivative 3 days]
IPTCC [Mean]
Emergency (site 2) "hyperthermia" % of sojourn [Minimum]
Emergency (site 2) "hyperthermia" % of sojourn [Mean]
IPTCC [Maximum]
% positive RT−PCR 60+ yo
New ICU [derivative 3 days]
Gironde RT−PCR 60+ yo [Maximum, derivative 3 days]
0.6
0.7
0.8
0.9
1.0
% selected

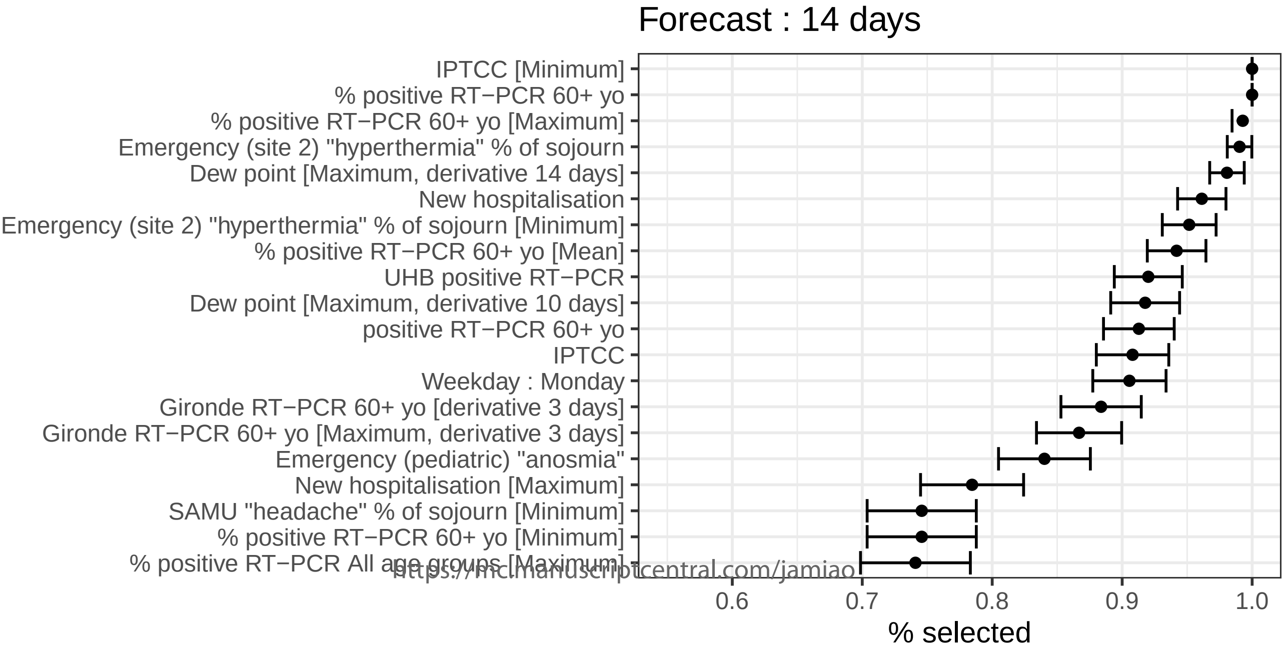

Forecast : 14 days
IPTCC [Minimum]
% positive RT−PCR 60+ yo
% positive RT−PCR 60+ yo [Maximum]
Emergency (site 2) "hyperthermia" % of sojourn
Dew point [Maximum, derivative 14 days]
New hospitalisation
Emergency (site 2) "hyperthermia" % of sojourn [Minimum]
% positive RT−PCR 60+ yo [Mean]
UHB positive RT−PCR
Dew point [Maximum, derivative 10 days]
positive RT−PCR 60+ yo
IPTCC
Weekday : Monday
Gironde RT−PCR 60+ yo [derivative 3 days]
Gironde RT−PCR 60+ yo [Maximum, derivative 3 days]
Emergency (pediatric) "anosmia"
New hospitalisation [Maximum]
SAMU "headache" % of sojourn [Minimum]
% positive RT−PCR 60+ yo [Minimum]
% positive RT−PCR All age groups [Maximum]
0.6
0.7
0.8
0.9
1.0
% selected

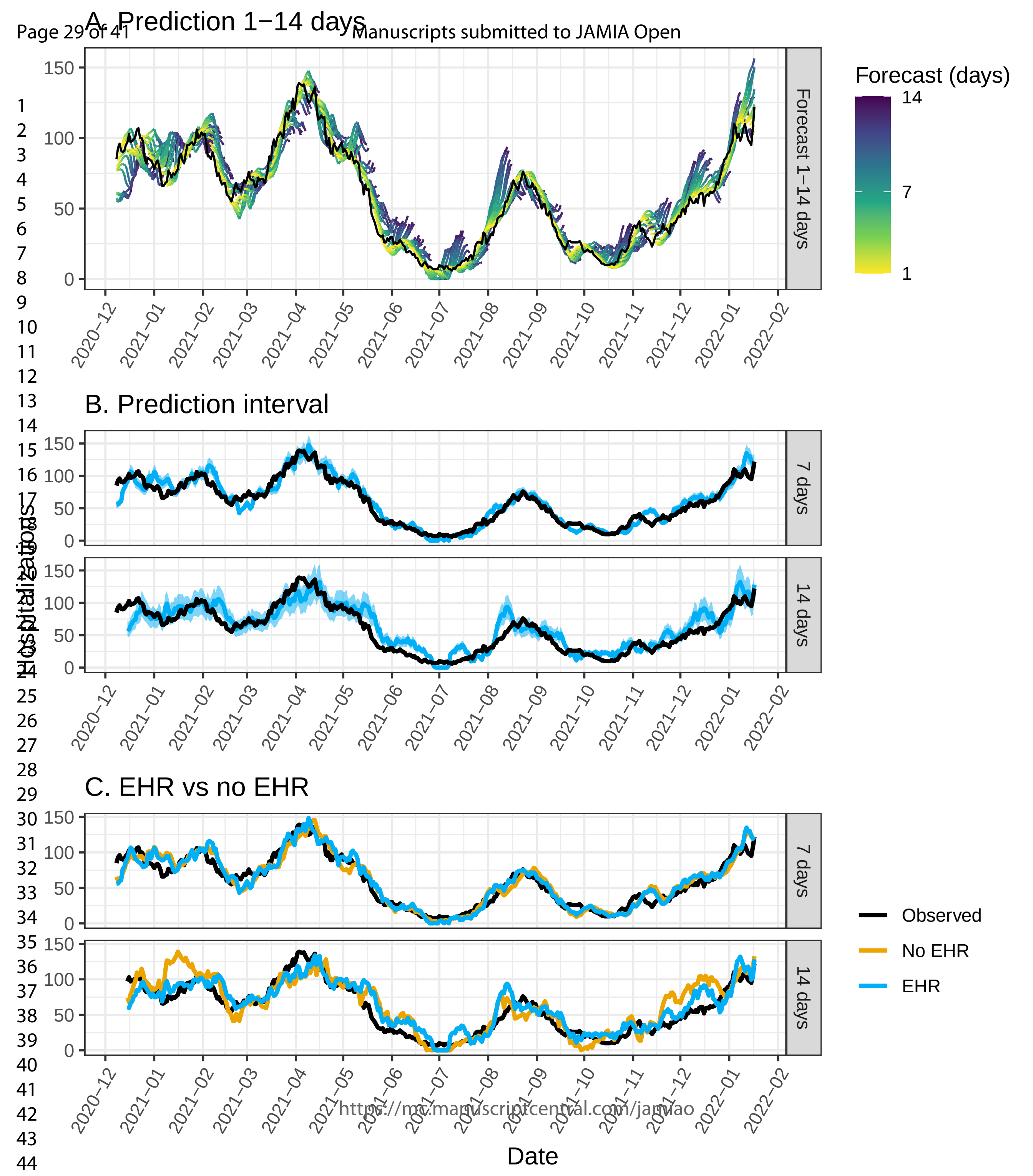

A. Prediction 1−14 days
Forecast 1−14 days
Forecast (days)
14
7
1
B. Prediction interval
7 days
14 days
C. EHR vs no EHR
7 days
14 days
Observed
No EHR
EHR
Hospitalizations
Date
2020−12
2021−01
2021−02
2021−03
2021−04
2021−05
2021−06
2021−07
2021−08
2021−09
2021−10
2021−11
2021−12
2022−01
2022−02

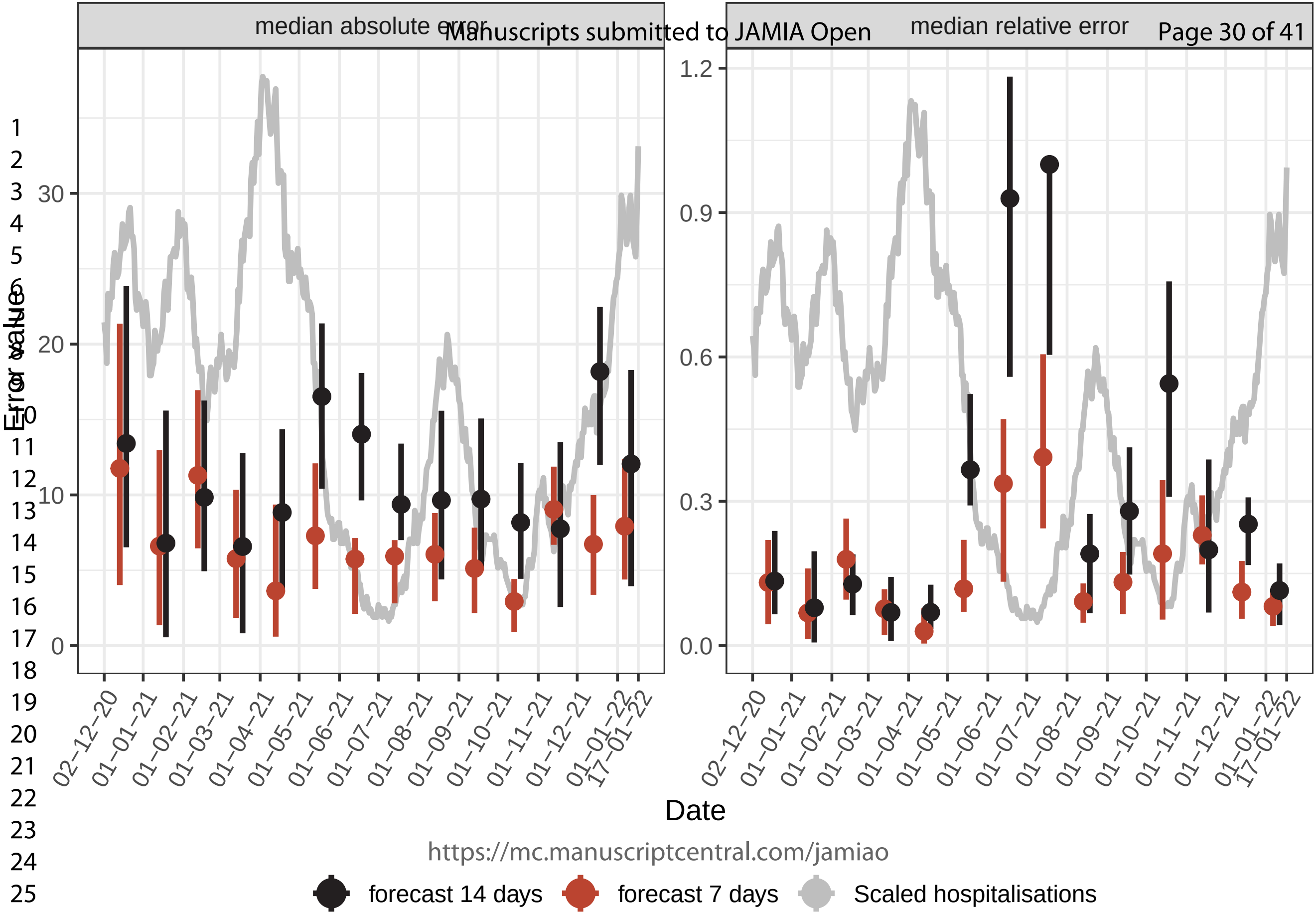

median absolute error
median relative error
Error value
Date
30
20
10
0
1.2
0.9
0.6
0.3
0.0
02−12−20
01−01−21
01−02−21
01−03−21
01−04−21
01−05−21
01−06−21
01−07−21
01−08−21
01−09−21
01−10−21
01−11−21
01−12−21
01−01−22
17−01−22
forecast 14 days
forecast 7 days
Scaled hospitalisations

## Supplementary Material

ooac086_Supplementary_Data

