# Supplementary material for: The benefit of augmenting open data with clinical data-warehouse EHR for forecasting SARS-CoV-2 hospitalizations in Bordeaux area, France
Source: JAMIA Open. 2022 Nov 11;5(4):ooac086. doi: 10.1093/jamiaopen/ooac086 (PMC9619837; doi:10.1093/jamiaopen/ooac086)
Supplement: ooac086_Supplementary_Data [file ooac086_supplementary_data.docx]

Supplementary material

**Bootstrap procedure for 95% confidence intervals:**

Bootstrap procedure was adapted to time series. For the forecast at 7 days, observations before a given date *d-*7 were sampled with replacement and the model was trained on those data. Then, the forecast was performed on the data of date *d* and the absolute and relative error were computed. This procedure was repeated 500 times at each date. For each date, we computed the 2.5% and the 97.5% absolute and relative error percentile. The confidence interval of median absolute and relative error was computed by taking the median of the 2.5% time series and the 97.5% time series. The same procedure was used for 14 days forecast.

**
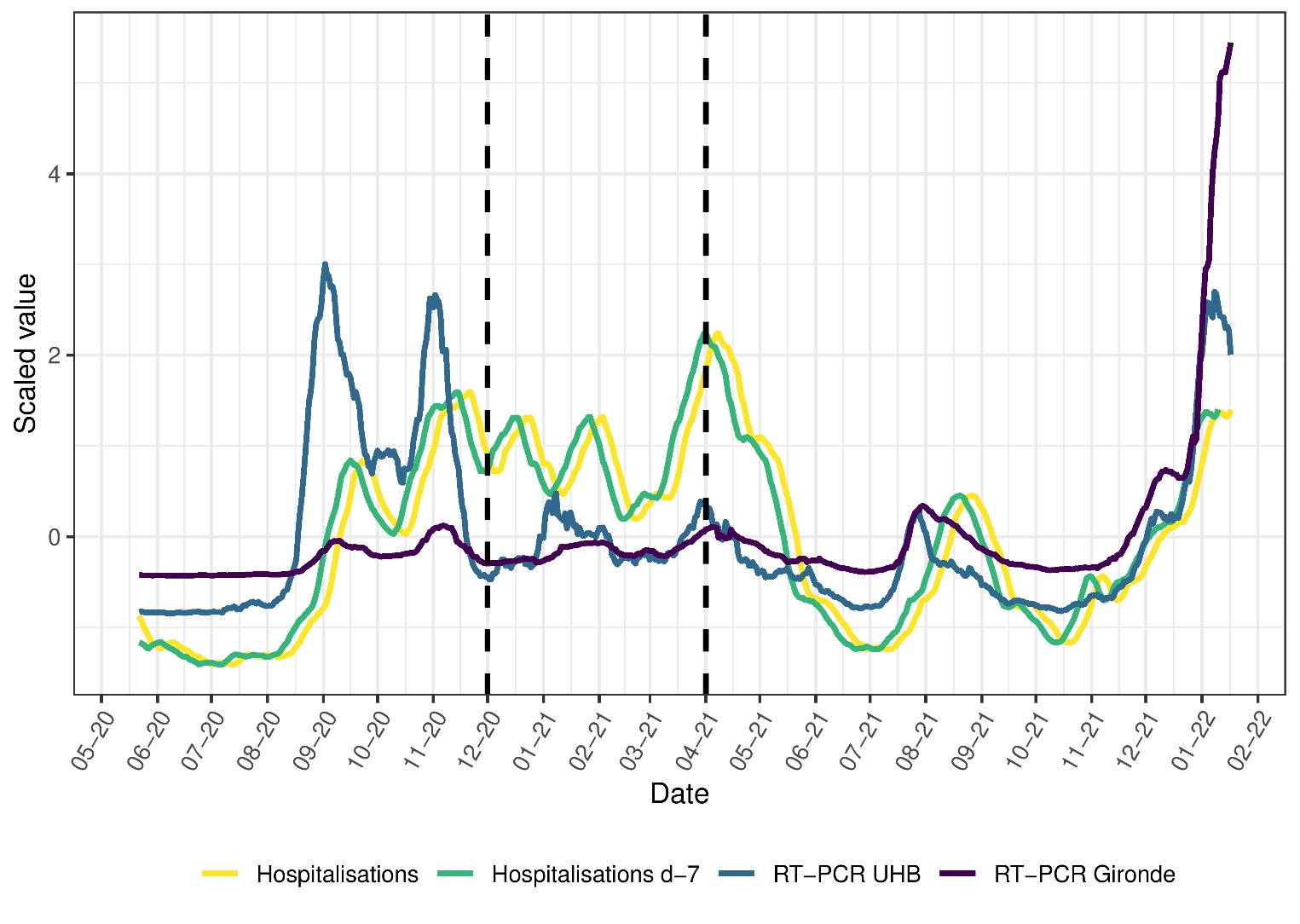
**

**Figure S1: SARS-CoV-2 positive RT-PCR and hospitalizations smoothed with 7 days moving average. Hospitalizations and RT-PCR values are scaled (i.e. mean-centered and divided by standard deviation) to be represented on the same figure.**

Figure S1 shows the number of positive RT-PCR performed at the Bordeaux hospital and the number of positive RT-PCR in the area (Gironde department) according to Santé Publique France database. The green curve corresponds to the observed number of hospitalizations with an anticipation of 7 days; this would be an ideal predictor. As shown on the figure, both RT-PCR in the Bordeaux Hospital and in Gironde have a dynamic similar to this predictor from June 2020 to December 2020. Unfortunately, the relationship between the two become less consistent beyond December 2020. For instance, Gironde RT-PCR are synchronous to hospitalization peak in April 2021 but do not anticipate it. In addition, the large increase of positive RT-PCR during the end of the summer 2021 is not associated with a similar increase of hospitalizations, which is probably due to vaccination. Those findings explain why the forecast of SARS-CoV-2 hospitalizations is difficult and why it is interesting to leverage different data sources to improve prediction capacity.

| **Table S1: Detailed features by data sources.** | |
| --- | --- |
| **Data source** | **Features** |
| Other | Department, Region, department population, day of the week |
| Gironde | Hospitalizations, RT-PCR (0-19, 20-59, >60 yo), positive RT-PCR (0-19, 20-59, >60 yo), proportion of positive RT-PCR (0-19, 20-59, >60 yo), number of people with one vaccine dose or more, majority variant |
| Bordeaux Hospital Hospitalizations and RT-PCR | Hospitalizations (number, admission and discharge), ICU (admission and discharge), RT-PCR (0-19, 20-59, >60 yo), positive RT-PCR (0-19, 20-59, >60 yo), proportion of positive RT-PCR (0-19, 20-59, >60 yo), intra hospital RT-PCR, extra hospital RT-PCR |
| Bordeaux Hospital Ambulance service and Emergency units (pediatric, Pellegrin, St-André units) | Number and proportion of emergency service sojourn or ambulance service call with mention of the following concepts: “headache”, “diarrhea”, “fever”, “covid-19 symptoms”, “ageusia”, “dyspnea”, “cough”, “hyperthermia”, “covid-19”, “anosmia”, any of the previous terms. |
| Weather | Temperature, precipitation, relative humidity, absolute humidity, IPTCC index, wind speed, dew point |

| **Table S2: Forecast performance by data source. Result from the additional analysis considering Bordeaux Hospital as an additional unit among other French department.** | | | | |
| --- | --- | --- | --- | --- |
| **Forecast** | **Data** | **MAE [95% CI]** | **MRE [95% CI]** |  |
| 7 days | Hosp + RT-PCR | 9·25 [8·30 ; 10·21] | 0·213 [0·196 ; 0·236] |  |
| 7 days | Hosp + RT-PCR + Weather | 8·84 [8·06 ; 9·98] | 0·206 [0·192 ; 0·231] |  |
| 7 days | Hosp + RT-PCR + Weather + Variants + Vaccine | 9·09 [7·98 ; 10·85] | 0·214 [0·183 ; 0·246] |  |
| 14 days | Hosp + RT-PCR | 15·07 [12·41 ; 17·38] | 0·308 [0·262 ; 0·356] |  |
| 14 days | Hosp + RT-PCR + Weather | 13·57 [10·60 ; 15·95] | 0·257 [0·194 ; 0·322] |  |
| 14 days | Hosp + RT-PCR + Weather + Variants + Vaccine | 17·27 [12·46 ; 22·02] | 0·370 [0·250 ; 0·472] |  |
| MAE: Median Absolute Error - MRE: Median Relative Error  Dpt-day: Bordeaux Hospital as an additional unit | | | | |

Table S2 shows the prediction performance of the model. It shows the addition of weather data did improve the forecast performance at both 7 and 14 days whereas the addition of variants and vaccine data deteriorated the performance.


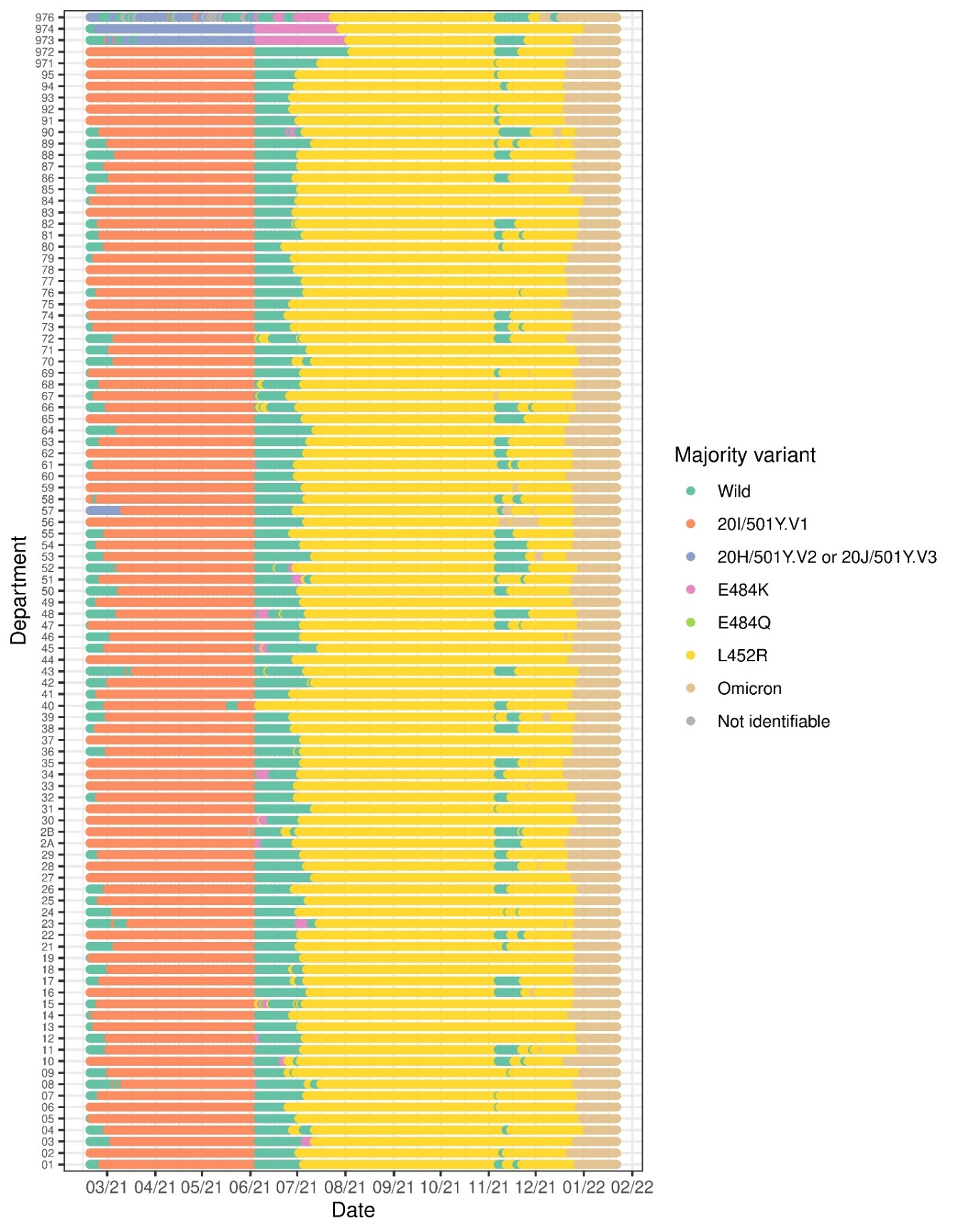


**Figure S2: Majority variant evolution by department. On the 2021-06-09, modality of variant data collection changes from variant identification (20I/501Y.V1, 20H/501Y.V2, 20J/501Y.V3) to mutation identification (E484K, E484Q, L452R). Since 01/11/2021, mutation related to Omicron variant are tested.**


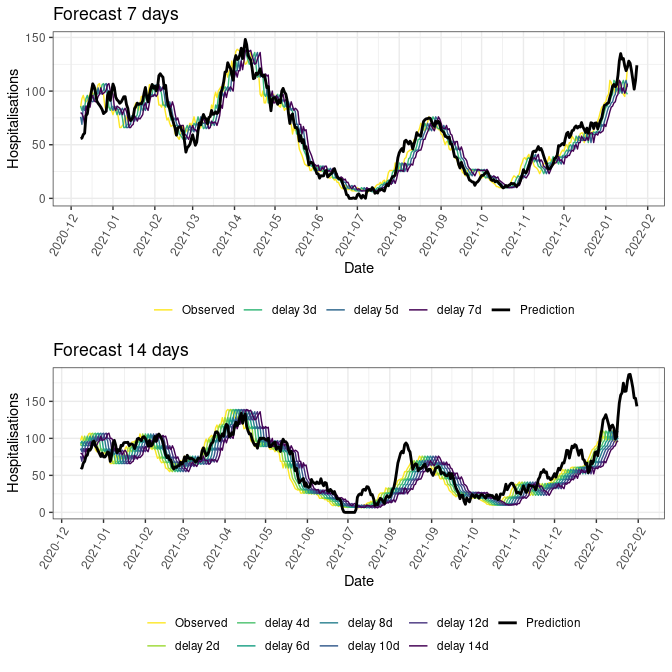


**Figure S3: Forecast prediction of the best model compared to observed values and delayed observed values. A perfect predictor would have black curve equals to yellow curve**


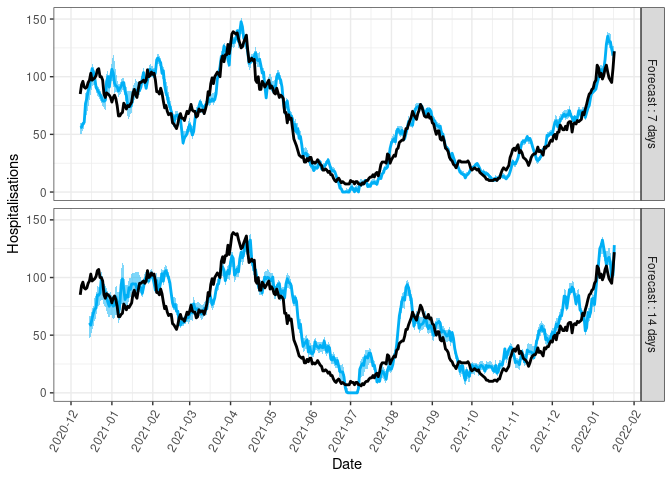


**Figure S4: Forecast of best model with 95% bootstrapped prediction intervals at 7 and 14 days.**

Figure S2 outlines the stability of the majority variant over time, which might explain why this feature does not improve the performance of the machine-learning model.

Figure S3 shows a different representation than the one proposed at figure 2.A focusing on the delay between the observed hospitalization evolution and the forecast. It shows at both 7 and 14 days that the forecast is mostly accurate. However, slope shifts are often predicted with a delay (e.g. April peak or February decrease).

Figure S4 shows the predictions of the best model with 95% bootstrapped prediction intervals. As shown on the figure, the intervals are too narrow and it justifies the use of the 20 and 40% margin at 7 and 14 days displayed at figure 2.B. Table S3 shows the prediction interval coverage of both methods, which are better even though far from 95% coverage for the 20/40% rule.

***
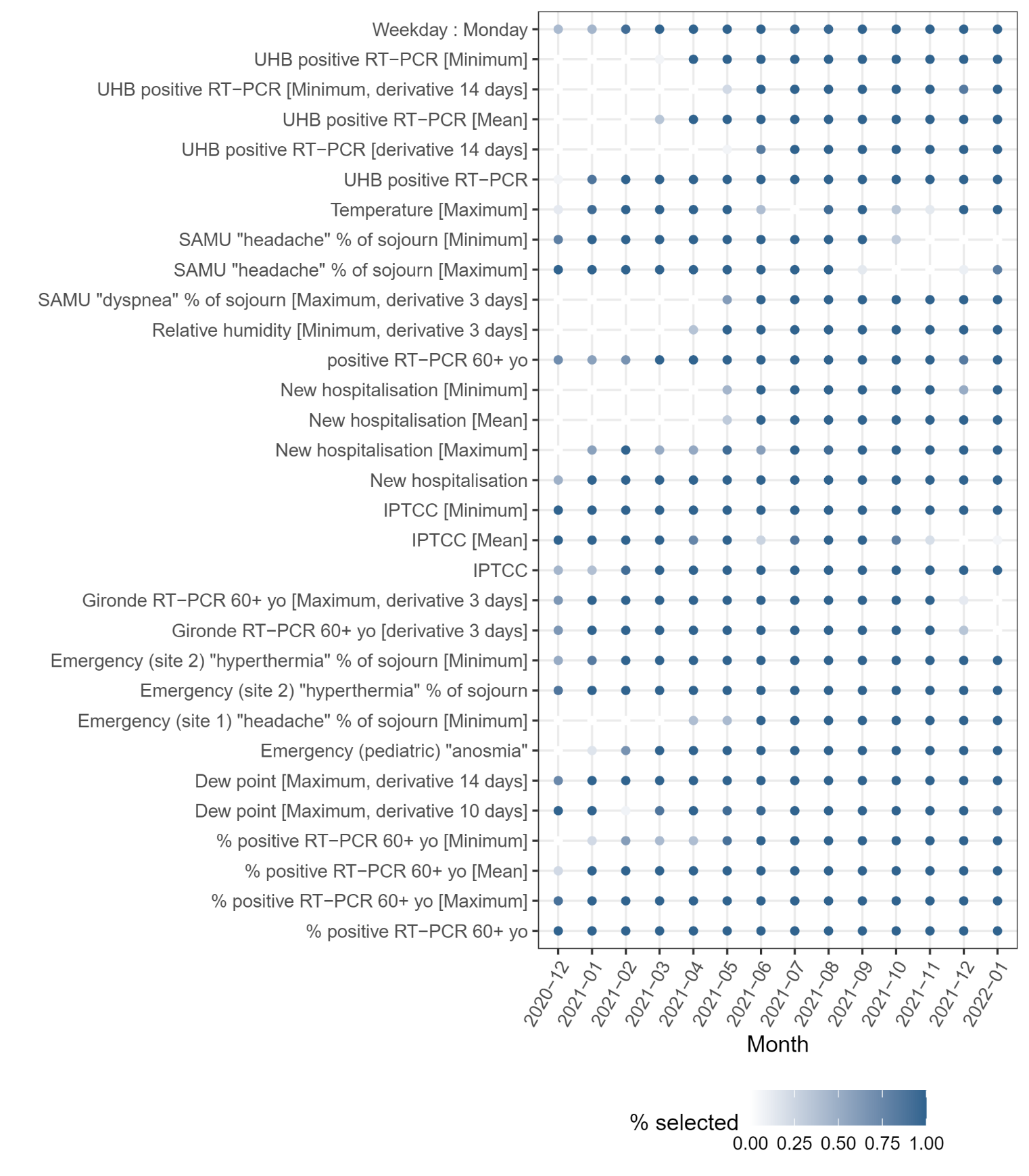
***

**Figure S5: Feature selection at 7 days by month. Each dot corresponds to the proportion of days with the feature selected by month. Only features selected every day of at least seven months are shown.**

***
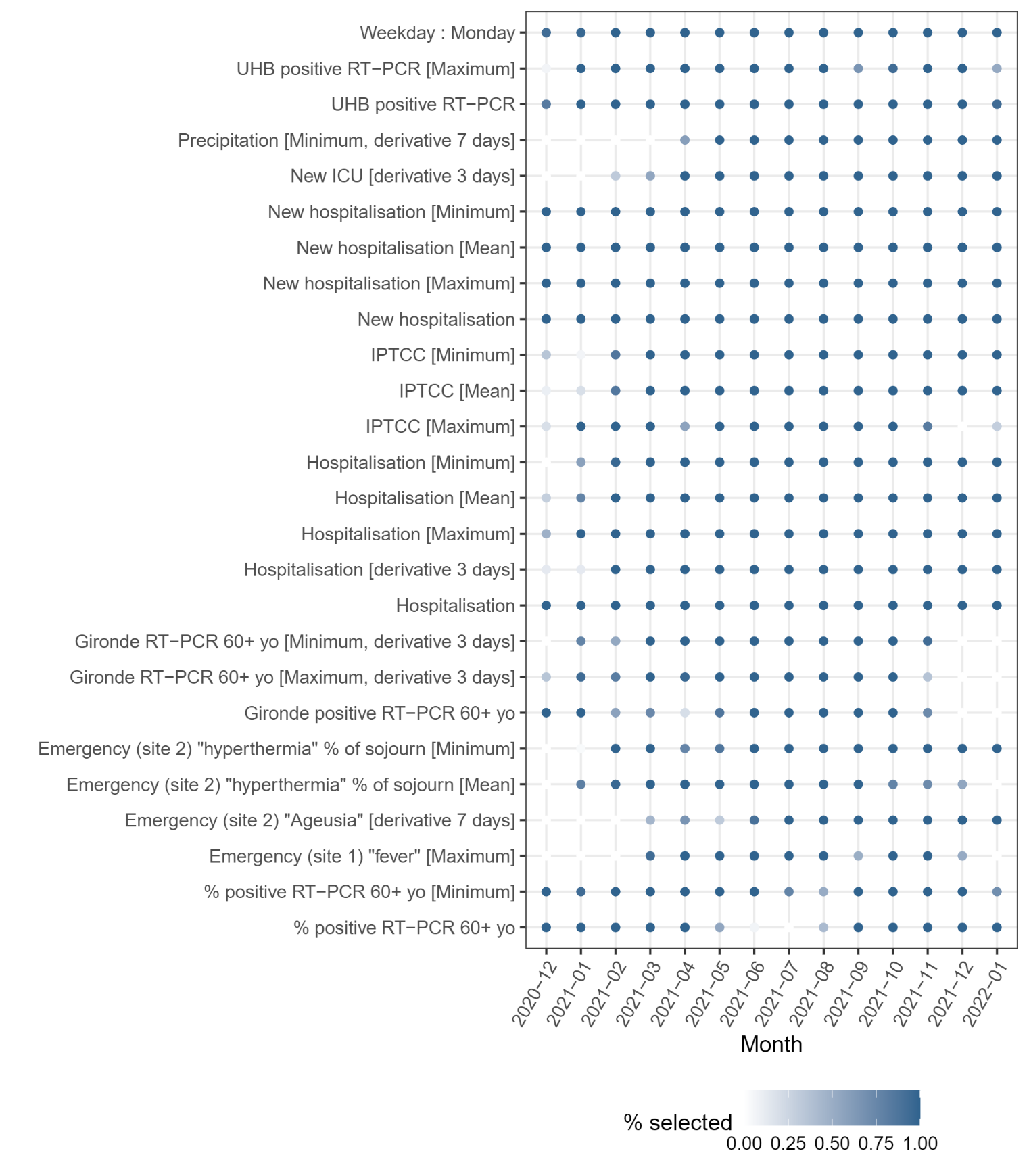
***

**Figure S6: Feature selection at 14 days by month. Each dot corresponds to the proportion of days with the feature selected by month. Only features selected every day of at least seven months are shown.**

Figure S5 and S6 shows the feature selection evolution over time for 7 and 14 days forecast, respectively. Only features selected every day of at least seven months ~~one month~~ are shown. The interpretation of those figures should be cautious as different features can represent the same concept (i.e. UHB positive PCR is both represented by its ~~minimum, its mean~~ maximum over the last 7 days and its value).

| **Table S3 : 95% prediction interval coverage by bootstrap and add hoc rule (20% at 7 days and 40% at 14 days). Results by time period.** | | | |
| --- | --- | --- | --- |
| **Forecast** | **Period** | **Bootstrap** | **Add hoc (20%-40%)** |
| 7 days | [2020-12-01 ; 2021-01-01) | 0·226 | 0·323 |
| 7 days | [2021-01-01 ; 2021-03-01) | 0·339 | 0·508 |
| 7 days | [2021-03-01 ; 2021-05-01) | 0·492 | 0·721 |
| 7 days | [2021-05-01 ; 2021-07-01) | 0·246 | 0·180 |
| 7 days | [2021-07-01 ; 2022-09-01) | 0·339 | 0·419 |
| 7 days | [2021-09-01 ; 2022-11-01) | 0·377 | 0·393 |
| 7 days | [2021-11-01 ; 2022-01-11) | 0·169 | 0·394 |
| 7 days | **Total** | 0·315 | 0·426 |
| 14 days | [2020-12-01 ; 2021-01-01) | 0·548 | 0·677 |
| 14 days | [2021-01-01 ; 2021-03-01) | 0·508 | 0·831 |
| 14 days | [2021-03-01 ; 2021-05-01) | 0·328 | 0·803 |
| 14 days | [2021-05-01 ; 2021-07-01) | 0·049 | 0·016 |
| 14 days | [2021-07-01 ; 2022-09-01) | 0·226 | 0·435 |
| 14 days | [2021-09-01 ; 2022-11-01) | 0·246 | 0·311 |
| 14 days | [2021-11-01 ; 2022-01-11) | 0·172 | 0·594 |
| 14 days | **Total** | 0·276 | 0·511 |


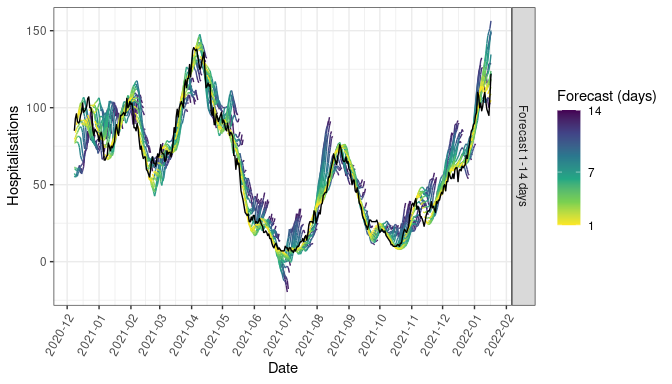


**Figure S7: Best model forecast from 1 to 14 days. Each string corresponds to the forecast from one (yellow) to 14 days (purple) at each day. Negative values are not set to zero.**

Figure S7 shows the forecast without setting negative values to zero. Negative predicted values occurred between the 2021-06-25 and 2021-06-30 for the 7 days forecast and between the 2021-06-15 and the 2021-07-06 for the 14 days forecast.
